# How effective and fair is user fee removal? Evidence from Zambia using a pooled synthetic control

**DOI:** 10.1002/hec.3589

**Published:** 2017-10-16

**Authors:** Aurélia Lépine, Mylène Lagarde, Alexis Le Nestour

**Affiliations:** ^1^ London School of Hygiene & Tropical Medicine London UK; ^2^ University of Otago Dunedin New Zealand

**Keywords:** financial protection, universal health coverage, user fees, Zambia

## Abstract

Despite its high political interest, the impact of removing user charges for health care in low‐income settings remains a debatable issue. We try to clear up this contentious issue by estimating the short‐term effects of a policy change that occurred in 2006 in Zambia, when 54 of 72 districts removed fees. We use a pooled synthetic control method in order to estimate the causal impact of the policy on health care use, the provider chosen, and out‐of‐pocket medical expenses. We find no evidence that user fee removal increased health care utilisation, even among the poorest group. However, we find that the policy is likely to have led to a substitution away from the private sector for those using care and that it virtually eliminated medical expenditures, thereby providing financial protection to service users. We estimate that the policy was equivalent to a transfer of US$3.2 per health visit for the 50% richest but of only US$1.1 for the 50% poorest.

## 1 INTRODUCTION

In the past two decades, several countries have taken steps to removing user charges for some or all curative care services (Yates, 2009), embracing the idea that user fees “deter people from using health services and cause financial stress” (World Health Organisation, 2010). Such decisions were motivated by the observation that user fees can reduce utilisation of care (Burnham, Pariyo, Galiwango, & Wabwire‐Mange, 2004; Deininger & Mpuga, 2005), in particular for poorer population groups whose demand for care is more price elastic (Gertler, Locay, & Sanderson, 1987; Gilson, 1997; Sauerborn, Nougtara, & Latimer, 1994). In Zambia, user fee removal was justified on the grounds that user fees appeared to decrease equity of access to health care and increase poverty (Kahenya & Lake, 1994; Masiye, Seshamani, Cheelo, Mphuka, & Odegaard, 2005; Sukwa & Chabot, 1997).

Some researchers have pointed that removing fees may not necessarily have the beneficial effects one could hope for (Gilson & McIntyre, 2005; McPake, Brikci, Cometto, Schmidt, & Araujo, 2011). Although removing fees has the potential to improve service coverage and access, hasty politically driven decisions with no prior preparation can lead to unintended effects, including quality deterioration due to lack of funds, excessive demands on health workers, and depletion of drug stocks (Gilson & McIntyre, 2005). In addition, the positive effects of removing user fees depend on the determinants of the demand for health care. Economic theory indicates that removing fees increase utilisation if fees represent a significant financial hurdle for households to access care. However, if other factors such as distance to facilities or limited perceived benefits of health care are the main drivers behind low utilisation, removing financial barriers may have a more limited impact.

Some early evidence of the effects of user fee removal in several sub‐Saharan countries suggested that utilisation of health care services would grow after fees were removed (Lagarde & Palmer, 2008). A more recent review of the evidence on maternal services reached similar optimistic conclusions (Hatt, Makinen, Madhavan, & Conlon, 2013).

However, both reviews underline the weakness of existing studies, which relied mostly on poor quality routine data and failed to provide a robust identification of the causal impact of the policy (Lagarde & Palmer, 2008). Recent studies have provided more robust evidence of the effects of free curative care through the (quasi) randomised introduction of health insurance, and their conclusions are less optimistic. 1Although from a theoretical perspective the two interventions are equivalent (no direct cost for using health care services if the insurance provides a full third‐party reimbursement), in practice, they have important differences. Insurance schemes often limit the number and type of health care providers that can be chosen by members, in a way that user fee removal does not. And there is also evidence that even when they are insured, more disadvantaged groups are likely to claim their benefits and use health insurance less (Devadasan, Criel, Van Damme, Ranson, & van der Stuyft, 2007) whereas user fee removal does not present any administrative obstacle to anyone. Using the phased randomised implementation of a social health insurance in Mexico, King et al. (2009) found no increase in the use of health care services by insured individuals, even though free care drastically reduced their medical expenses. In Gujarat, a programme offering free deliveries to poor women in private facilities was not associated with a change in the probability of institutional delivery (Mohanan et al., 2014). Slightly more positive results emerged from a randomised controlled trial in Ghana (Powell‐Jackson, Hanson, Whitty, & Ansah, 2014), where free care resulting from the introduction of health insurance led to a small increase of utilisation of service (3.7 percentage points).

In this paper, we contribute to this debate by presenting new evidence from Zambia where fees were removed in primary care facilities in 54 of the 72 districts in 2006. Using the synthetic control method (Abadie & Gardeazabal, 2003; Cavallo, Galiani, Noy, & Pantano, 2013), we estimate the causal impact of user fee removal on health‐seeking behaviours, provider choice, and medical out‐of‐pocket (OOP) expenditures in the general population and explore some heterogeneous effects depending on income level.

The remainder of the paper is organised as follows. In Section 2, we describe the background and study setting. Section 3 describes the data, and Section 4 the empirical approach adopted. Section 5 presents the results and Section 6 the robustness checks. Section 7 discusses the findings and concludes.

## 2 BACKGROUND

### 2.1 Health‐seeking behaviours and user charges in Zambia before 2006

From 1964 to 1991, the government of Zambia provided health care services for free. In 1991, user fees were introduced to raise additional income to improve quality of services (avoid drug stock‐outs and increase staff motivation thanks to salary top‐ups) and greater accountability to the local communities. 2Two reports showed that user fee's introduction led to a decrease in utilisation (Kahenya & Lake, 1994; Sukwa & Chabot, 1997). User fees in primary care consisted of a flat consultation fee covering consultation and drugs, set by each district according to the ability to pay of the population (Carasso et al., 2010). The typical level of fees at primary care level could be considered as relatively low (McPake et al., 2011), typically between 500 to 1,000 Zambian Kwachas (about US$ 0.14 to US$ 0.27 in 2006; Carasso et al., 2010) or 5% to 10% of the equivalent of a day's average GDP per capita in 2006. Several categories of individuals were exempted from paying fees: patients under 5 and over 65 years old, pregnant women, those suffering from certain diseases (e.g., HIV/AIDS and TB), and indigents (identified by local communities). In practice, children and elderly people made up the majority of exemptions (respectively, 66% and 7% of all exemptions in 1998).

With rapid economic growth and the development of its public health care system, Zambia experienced a sharp increase in the proportion of sick individuals seeking modern care, from 35.89% in 1998 (Central Statistical Office, 1999) to 57.31% in 2004 (Central Statistical Office, 2005). In 2004, 56% of individuals reporting an episode of illness sought modern care, 17% did not do anything, and 27% chose self‐medication, usually meaning that they went to drug stores to obtain over‐the‐counter medicines. Among those who sought care, 82% of individuals went to a government facility, 8% to mission providers, 6% to private providers, 1% to traditional practitioners, and 3% to other providers.

### 2.2 The 2006 policy change

On January 13, 2006, the Zambian president announced that user fees in primary health care were to be removed in rural areas as a first step towards universal access for all (Carasso et al., 2010). The policy would apply to publicly funded facilities, which included both government‐run as well as mission facilities. Facilities could still charge two categories of patients: those coming from outside of the catchment area and foreigners. 3The policy change was later scaled up to other areas. In January 2007, user fees were removed in all public health facilities located in the peri‐urban areas of the remaining 18 urban districts. Finally, in January 2012, user fees were removed everywhere else.


Following the presidential announcement, a directive was sent to all districts in March 2006 stating that the policy would apply to all primary health care facilities located in rural areas, everywhere in the country. However, due to multiple challenges to clearly define rural areas, the government changed the definition of the policy at the last moment, and on April 1, 2006 all primary care facilities located in the 54 districts designated as “rural” according to the local government classification were asked to remove user fees. Some confusion ensued at the beginning of the policy change, with local authorities not always clear about the remits of the policy (Carasso et al., 2010).

To help prevent potential negative effects due to the loss of user fee revenue at facility level, facilities would be compensated through an earmarked monthly grant, to be paid by each district. These compensation grants were loosely linked to actual utilisation of health care services because they were based on projected income loss calculated by each district, based on 2005 routine data (Government of the Republic of Zambia, 2006). The payment of these grants, funded by a bilateral donor, ended up being compromised by several factors. Essentially, the funds were released to the Zambia Treasury only in August 2006, and they only reached rural districts between December 2006 and March 2007. Furthermore, in the absence of clear guidelines on how to use this additional funding, district authorities followed different approaches: some facilities received monthly payments, whereas others received lump sum payments or no grant at all (Carasso et al., 2010).

In addition to the lack of replacement funds, the 2006 policy change was introduced in a particularly challenging year for funding to primary health care facilities, as there was a 40% reduction in funding to district primary health care (Government of the Republic of Zambia, 2007). In effect, this meant that primary care facilities experienced a double loss in revenue, from the district basket as well as from user fees.

Due to these implementation challenges, user fees were not effectively abolished for everyone in rural districts. According to national household survey data, 6 months after the official introduction of the free care policy, 29% of patients aged between 5 and 65 years living in rural districts were still paying for receiving care in a government‐run or mission health centre. Yet, this still represented a sharp decrease compared to 2004, where 64% of the same population would be charged for health care services.

### 2.3 Anticipated effects

Basic economic theory suggests that a decrease in price will increase the demand for health care services through an income effect and a substitution effect. In the Zambian context, the income effect would allow more people to use public health care services, and the change in the relative prices of different care‐seeking options would lead to a substitution away from private health providers (Gertler & Gaag, 1990). However, these effects would only occur if the demand for public health care services is sensitive to price and if the perceived quality of care remains unchanged. Both assumptions are potentially problematic in the case of Zambia. There are reasons to believe that changes in prices happened in conjunction with changes in quality of care and that the perceived quality of care diminished in the public sector. Indeed, in Zambia, the health financing reform took place against the backdrop of a critical shortage of health workers, which affected particularly remote and rural areas (Carasso, Lagarde, Cheelo, Chansa, & Palmer, 2012; McPake et al., 2013). As a result, it is possible that populations in rural areas would expect relatively low quality of services in the public sector after fees were removed, mitigating their valuation of these free services.

### 2.4 Existing evidence

There have been a few studies looking at the impact of removing user fees in Zambia. Several have been descriptive, documenting the implementation of the policy and the way health care providers or community members perceived it (Carasso et al., 2012; Hadley, 2011; Masiye, Chitah, Chanda, & Simeo, 2008).

Three studies used routine facility data to investigate the policy impact (Chama‐Chiliba & Koch, 2016; Lagarde, Barroy, & Palmer, 2012; Masiye, Chitah, & Mcintyre, 2010). Although their findings are not directly comparable due to the different scope and type of data and methods used, those studies find mixed evidence regarding the effect of user fee removal on the demand for health services. Using an interrupted time series approach, Lagarde et al. (2012) estimate a 40% increase in the volume of outpatient visits 6 months after the policy change in a subset of 17 rural districts, with that effect flattening out over time. Masiye et al. (2010) compare trends in the volume of quarterly visits before and after the policy change and find that they increased in rural districts but not in urban districts, or for children under 5. Meanwhile, Chama‐Chiliba & Koch, 2016 use birth histories obtained from household data and find that the policy did not have any significant increase on deliveries in public health facilities.

These studies may not have detected the causal impact of the policy for two reasons. First, the identification strategies used are problematic. Masiye et al. (2010) caution that they cannot evaluate the causal impact of the policy, but rather some changes in trends. The validity of the interrupted time series approach used by Lagarde et al. (2012) hinges on the assumption that no other concurrent factor may have affected the outcome of interest over the study period. Based on reports and anecdotal evidence of the policy change and the human resources crisis over the period, this assumption is debatable. Finally, Chama‐Chiliba & Koch (2016) rely on a difference‐in‐difference identification strategy, but fail to provide evidence of the parallel trend assumption.

Second, two of these studies rely on facility register data that have at least two limitations. First, they suffer from measurement errors, including not at random missing data and obvious inaccuracies, as indicated by Lagarde et al. (2012). Second, these routine data report the volume of outpatient consultations, which do not differentiate between unique visits of different patients and multiple visits by the same patients. In other words, the increase in the volume of consultations could be explained by an increase in the frequency of visits of “current” users and not by an increase in utilisation by new users.

## 3 DATA

Data on the study outcomes come from the Living Conditions and Monitoring Surveys (LCMS), a repeated cross‐sectional household survey designed to provide the basis for comparison of poverty estimates in Zambia over time. Each survey includes detailed information about health‐seeking behaviours, as well as a variety of socio‐economic variables on a nationally representative sample of the population.

We make use of all surveys we could pretreatment and posttreatment: three waves of the survey pretreatment (1998, 2002, and 2004) 4Due to a change in administrative definition of districts, we could not use surveys that took place before 1998. and one survey posttreatment (2006). For that last survey, the data collection occurred over October–November 2006, meaning that we observe the variables of interest 6 months after the policy change. Summary statistics on the outcome measures used before and after the removal of user fees are shown in Panel A of Table 1. In addition to information on study outcomes, we exploited data on a broad range of socio‐demographic characteristics as detailed in Panel B of Table 1.

**Table 1:** Mean in outcomes and covariates

	1998 (*n* = 72)		2002 (*n* = 71)		2004 (*n* = 72)		2006 (*n* = 72)	
	Urban	Rural	Urban	Rural	Urban	Rural	Urban	Rural
**Panel A**								
% seeking modern care when ill	0.358	0.310	0.505	0.520	0.565	0.548	0.564	0.584
% choosing a government or mission facility^a^	0.778	0.898	0.829	0.910	0.862	0.905	0.832	0.957
% buying drugs in the private sector^a^			0.129	0.019	0.114	0.022	0.090	0.033
Log of deflated OOP^a^	6.703	6.522	5.967	4.547	6.149	5.374	6.088	3.111
**Panel B**								
Proportion of male	0.496	0.492	0.489	0.491	0.498	0.494	0.490	0.487
Median age	20.060	19.761	21.162	20.448	21.213	20.488	21.051	19.966
Median log of total expenditures in adult equivalent	13.781	13.268	13.487	13.202	13.483	13.069	12.904	11.752
Median household size	7.039	6.565	6.287	6.496	6.737	6.874	6.107	5.829
Proportion of rural	0.311	0.885	0.303	0.905	0.299	0.807	0.260	0.798
Median distance to health facility (km)	1.580	5.183	1.723	5.784	1.853	4.289	1.104	5.573

*Note*. All values are representative at the national level as the district panel data were constructed based on sampling weights. In 2002, there were only 71 districts because there was no one who sought care when reporting illness in one district. OOP = out‐of‐pocket.

a Proportion of individuals seeking modern care.

We investigate the effect of the policy on four outcomes. First, we look at health care utilisation, defined as whether an individual who reported an illness episode the last 2 weeks sought modern formal care—this excludes consultation of traditional or church healers and self‐medication but includes private, government, and mission facilities. Second, we consider the choice of provider of individuals seeking modern care. Specifically, we look at the proportion who went to any government‐funded primary care facility 5This is defined as an individual declaring they went to seek care in a government or a mission facility. to test whether the policy led to a substitution away from private‐for‐profit providers. Third, to estimate the effect of the policy on financial protection, we consider health care expenditures incurred by individuals at the point of care, defined as the amount of OOP medical expenses (deflated and expressed in Kwachas 2006). Finally, we consider a potential unintended consequence of the policy, understood as something that was not meant by the policy change. Specifically, we look at the proportion of individuals who sought care and had to purchase drugs from a private pharmacist. This is meant to detect whether increased utilisation of public facilities led to drug stock‐outs, a sign of poor quality of care, and a cause for additional expenditures incurred elsewhere.

## 4 EMPIRICAL STRATEGY

### 4.1 The synthetic control method

The staggered implementation of the policy change creates a natural experiment to analyse the effects of user fee removal. The 54 districts implementing the policy change in April 2006 are treatment units whereas the remaining 18 urban districts where fees are still charged constitute the donor pool (i.e., the comparison group). We exploit the fact that district classification was highly arbitrary because districts containing a city or a municipality were classified urban, whereas the other districts were classified rural. Nevertheless, looking at the distribution of the proportion living in rural areas (see Appendix S1), we see that on average, 40% of households living in an urban district live in a rural area and that there exist some highly rural areas among the urban classified districts, which provides reassurance about the choice of urban districts as reasonable controls for rural districts.

**Figure 1:**
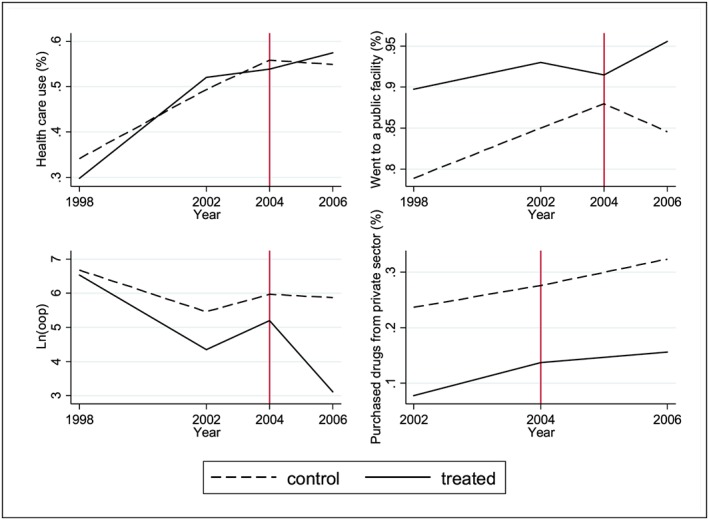
Trends in outcomes [Colour figure can be viewed at http://wileyonlinelibrary.com]

However, as suggested by the graphs in Figure 1, we ruled out a simple difference‐in‐differences (DiD) approach because the pre‐intervention outcome trends in the control and treatment groups are not parallel for most outcomes (specifically health care utilisation and health provider choice). 6Results are presented as a robustness check.


An alternative method to estimate causal effects of a policy affecting one or more units is the synthetic control method (Abadie, Diamond, & Hainmueller, 2010; Abadie & Gardeazabal, 2003; Cavallo et al., 2013). This method involves constructing a counterfactual for the treated group (rural districts) by taking a weighted average of the available control units (urban districts), where a higher weight is given to control units that are more similar to the treated unit. This synthetic twin is created to follow the same pattern than the treated unit in the pretreatment period so that it can be used as a counterfactual after the policy implementation.

Additionally, the synthetic control is built by using the observable characteristics in all the pretreatment years. Unlike matching estimators, the idea behind the synthetic control is that a combination of control units provides a better comparison for the treated unit than a single unit alone. Additionally, the synthetic control is built by using the observable characteristics in all the pretreatment year allowing the effects of the unobserved heterogeneity in the outcome to vary with time (Abadie et al., 2010).

Using survey sampling weights, we estimate mean values for all outcomes and independent variables at the district level. Because they already benefited from free care before 2006, we exclude from the analysis individuals aged less than 5 and more than 65 years old in each survey wave. Based on available data, it is not possible to exclude other exempted groups but they represent a small proportion of all exemptions, and the exemption rules remain the same over the period of interest. We obtain a panel of 72 districts observed over three pretreatment periods (1998, 2002, and 2004) and one posttreatment period (2006).

Following Abadie et al. (2010), let $Y_{\mathrm{it}}^{N}$ be the outcome observed for district *i* at time *t* in the absence of intervention, for districts *j* = 1, …, *J* and time periods *t =* 1, …, *T*; and let the treated district *i =* 1 be the only one exposed to the intervention only after *T*_0_ (with 1 ≤ *T*_0_ < *T*). Let $Y_{\mathrm{it}}^{I}$ be the outcome that would be observed for district *i* at time *t* if district *i* is exposed to the intervention. The effect of the intervention for district *i* at time *t* > *T*_0_ can be defined as $\alpha_{\mathrm{it}}=Y_{\mathrm{it}}^{I}\;-\;Y_{\mathrm{it}}^{N}$. Because $Y_{1t}^{I}$ is observed for a treated district *i* = 1, to estimate *α*_1*t*_, we just need to estimate $Y_{1t}^{N}$.

To construct the counterfactual outcome in the treated district in the absence of the intervention $\;Y_{1t}^{N}$, the synthetic control method seeks an optimal vector of weights W* $={\left(\omega_{2}^{*},\ldots ,\omega_{j+1}^{*}\right)}^{\prime }$ chosen to minimise the distance between pre‐intervention characteristics and outcomes for the treated districts (*X*_1_) and for the control districts (*X*_0_; Abadie et al., 2010). Using these weights, the synthetic control for unit *i* is given by

$${\hat{Y}}_{1t}^{N}=\sum_{j=2}^{j=j+1}w_{j}^{*}Y_{\mathrm{jt}}\;\mathrm{for}\;t\;>\;T_{0}.$$

And therefore, the effect of the intervention for district *i* = 1 is ${\hat{\alpha }}_{1t}=Y_{1t}-{\hat{Y}}_{1t}^{N}$.

Formally, if *W* = (*ω*_2_, …, *ω*_*j* + 1_)^′^ is a vector of weights such that *w*_*j*_ ≥ 0 for *J* = 2, …, *J* + 1 and *w*_2_ + … + *w*_*j* + 1_ = 1, then the optimal vector W* is chosen to minimise the distance between *X*_1_ and *X*_0_*W*, measured thanks to the following:

$$\left\|X_{1}-X_{0}W\right\|v=\sqrt{X_{1}-X_{0}W){}^{\prime }V\left(X_{1}-X_{0}W\right)},$$

where *V* is a diagonal positive semidefinite identity matrix of dimension (K × K) that minimises the root mean squared prediction error (RMSPE), that is, the average of the squared discrepancy between the level of outcomes in the treated units and in their synthetic control counterpart in the pretreatment periods.

In addition to the pre‐intervention outcomes levels, here, we include the following district level covariates in both *X*_1_ and *X*_0_ 7Note that those covariates were chosen to be included because the RMSPE is minimised under this specification. Given the nonparametric nature of the synthetic control method, we have run a sensitivity analysis on the covariates to include and the model associated with the smallest RMSPE was selected. More specifically, we estimated a model with no covariate as well as another model that includes province dummies and district population size. Although the coefficients obtained were very close, those models lead to a higher RMSPE and higher confidence intervals and hence are not presented in the paper but are available from authors upon request.: proportion of male, proportion of households living in a rural area, median age, median household income, median household size, and median distance to the health facility.

Unlike the seminal case presented by Abadie et al. (2010) where there was only one treated unit, here, the policy change affected 54 rural districts. This implies that we compute ${\hat{\alpha }}_{\mathrm{it}}$ for each of the 54 treated districts. Then, to obtain a national‐level estimate of the policy effect, we take the average of 54 ${\hat{\alpha }}_{\mathrm{it}}$, weighted by the district population size (alternative approaches are used as robustness checks).

Statistical significance of the estimated effect is determined by running placebo tests (Abadie et al., 2010; Abadie & Gardeazabal, 2003). Specifically, we apply the synthetic control method to every untreated district (urban districts) in our sample. This allows us to assess whether the effect estimated by the synthetic control for the treated districts is large relative to the effect estimated in untreated districts. The idea is that if the distribution of placebo effects yields many effects as large as the estimated effect, then it is likely that the estimated effect was observed by chance.

### 4.2 Choice of control districts

The selection of the units to include in the donor pool (i.e., the technical term for all potential control units in the synthetic method approach) is crucial in the synthetic control method. We should consider discarding districts from the donor pool whose outcomes may be affected by the policy change because this could lead to underestimating its effect for two reasons. First, because the counterfactual outcomes for each synthetic treated district will be constructed as a weighted average of the outcomes of control districts, if some control districts are contaminated by the policy change (and have outcome levels comparable to those in treated districts), the policy effect will be underestimated if these contaminated control districts are given a nonzero weight. Second, because the statistical significance of the policy effect is evaluated against the distribution of placebo effects, if control districts are somehow contaminated by the policy, this will compromise our ability to detect that the change in the treated districts was not obtained by chance.

**Figure 2:**
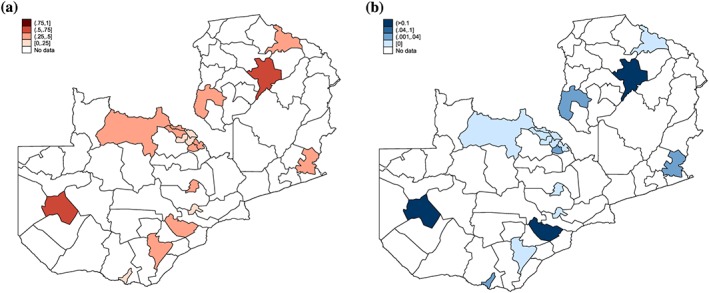
(a) Implementation in urban districts. (b) Proportion of urban populations who sought care in a rural district [Colour figure can be viewed at http://wileyonlinelibrary.com]

To choose the pool of donor districts, we have to consider two potential problems. First, due to the confusion around the implementation rules of the policy, some facilities located in the rural areas of urban districts may have wrongly decided to scrap user charges. Figure 2a shows a map of the proportion of patients from urban districts aged between 5 and 65 years who declared to have received free care. In two districts (Kasama and Mongu), more than half of the respondents received free care.

Second, although we assume that individuals seek care in the district where they live, people from urban districts could seek care in rural districts because facilities are closer or cheaper. On the basis of 1998 LCMS data, 8The only survey wave for which the information is available. we find that this issue is generally limited (less than 4% of the population of urban districts seeking care in rural districts—see Figure 2b), except in three districts (Mongu, Mazabuka, and Kasama) where respectively 25%, 18%, and 12% of the population sought care in rural districts.

On the basis of this, we construct a synthetic control for each of the 54 treated districts by using three alternative pools of control districts: (1) all 18 urban districts; (2) all but two districts (Kasama and Mongu) excluded because a significant proportion of the population received free care in 2006; and (3) all but three urban districts (Kasama, Mongu, and Mazabuka) where more than 10% of the population was declaring seeking care in a rural district in 1998.

## 5 RESULTS

### 5.1 Effects on access to modern care

Table 2 presents the estimated effects of the policy on the use of modern care, with the three alternative choices of control units.

**Table 2:** Effect of user fee removal on access to modern care

	(1)	(2)	(3)
**Panel A**			
Seeking care	**0.007**	**0.016**	**0.014**
90% CI	[−0.16, 0.13]	[−0.16, 0.13]	[−0.16, 0.13]
*N* treated	51	51	48
*N* placebo	18	16	15
**Panel B**			
Chose government or mission primary care provider	**0.016**	**0.087**	**0.080**
CI	[−0.19, 0.12]	[−0.19, 0.12]	[−0.17, 0.12]
*N* treated	53	53	53
*N* placebo	18	16	15

*Note*. Effects of the policy are reported in bold. The 90% CI in brackets reports the 5th and 95th percentile of the placebo test distribution. Specification (1) is estimated using all 18 urban districts as control districts. Specification (2) excludes two districts where more than 50% of the population was reported to have benefited from free care in 2006. Specification (3) excludes the previous two districts and a third one where more than 10% of the population sought care in a rural district in 1998.

The findings in Panel A show no evidence that the policy increased health care use. The estimated effects suggest that there was an increase in utilisation of modern care by 0.7 to 1.6 percentage points (Columns 1 and 2), which is not statistically different from zero. 9Placebo test graphs are presented in Appendix S2.


Panel B presents the estimated impact of the policy on the choice of provider, conditional on using modern care. The policy seems to have led to some substitution away from the private sector, as once we account for possible contamination, we find that the proportion of individuals who went to a public health facility increased by up to 8.7 percentage points. However, this effect is not statistically significant at the 10% level because it lies inside the placebo effect distribution. 10Placebo effects are presented in Appendix S2.


### 5.2 Effects on OOP expenditures

Turning to OOP expenditures (Table 3), the results indicate a significant and important impact of the policy, a reduction of OOP medical expenditures by 2.18 logarithm points, which is a 89% decrease 111−(exp(−2.177)) = 0.89 compared to 2004 expenditures (Column 1). This effect lies outside the “90% confidence interval” provided by the placebo effect distribution, meaning that the policy effect on financial protection is statistically significant at the 10% level. The reduction in OOP health expenses represents a saving during the last medical contact of US$2.3 (in 2006 US$) or 7% of the monthly adult equivalent expenditure. 12OOP's level in 2004 was Kwatchas 9481 or 2006 US$2.58.


**Table 3:** Effect of user fee removal on out‐of‐pocket health expenses

	(1)	(2)	(3)
Ln(oop)	**−2.177**	**−2.258**	**−2.279**
% change compared to 2004	***−89%***	***−90%***	***−90%***
CI	[−1.99, 1.56]	[−1, 1.56]	[−1, 1.56]
*N* treated	48	49	47
*N* placebo	18	16	15

*Note*. Effects of the policy are reported in bold and percentage change are reported in italics. The 90% CI in brackets reports the 5th and 95th percentile of the placebo test distribution. Specification (1) is estimated using all 18 urban districts as control districts. Specification (2) excludes two districts where more than 50% of the population was reported to have benefited from free care in 2006. Specification (3) excludes the previous two districts and a third one where more than 10% of the population sought care in a rural district in 1998.

### 5.3 Unintended effects

We now consider whether removing fees led to drug stock‐outs government and mission facilities, leading more patients to buy drugs from private facilities

The results (Table 4) show that the policy had a negative effect on the likelihood of buying drugs from the private sector; we estimate a decrease in the probability of buying drugs from the private sector between 1.7 and 6 points depending on the specification (Columns 3 and 1). However, these effects are not statistically significant and are consistent with the fact that drug shortages did not occur more in intervention districts.

**Table 4:** Effect on the proportion of individuals who bought drugs in private pharmacies

	(1)	(2)	(3)
Bought from a private drug provider	**−0.060**	**−0.043**	**−0.017**
CI	[−0.24, 0.19]	[−0.24, 0.19]	[−0.24, 0.19]
*N* treated	51	49	50
*N* placebo	18	16	15

*Note*. Effects of the policy are reported in bold. The 90% CI in brackets reports the 5th and 95th percentile of the placebo test distribution. Specification (1) is estimated using all 18 urban districts as control districts. Specification (2) excludes two districts where more than 50% of the population was reported to have benefited from free care in 2006. Specification (3) excludes the previous two districts and a third one where more than 10% of the population sought care in a rural district in 1998.

### 5.4 Factors affecting the impact of the policy

**Figure 3:**
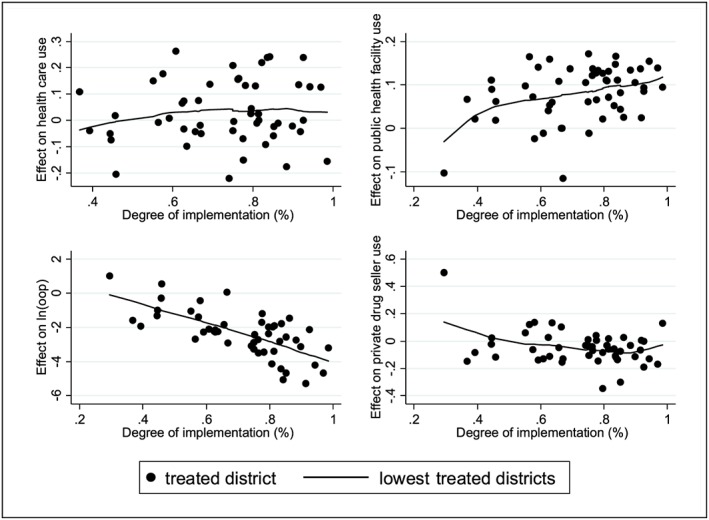
Relationship between the impact of the policy and its degree of implementation [Colour figure can be viewed at http://wileyonlinelibrary.com]

We now investigate whether chaotic implementation of the policy led to its lack of documented impact on health‐seeking behaviours. Figure 3 shows, for the four outcomes, the estimated effect against the degree of implementation of the policy in each district defined as the proportion of individuals not paying for primary care. These graphs suggest that although a better implementation of the policy is correlated with a greater use of publicly funded health facilities and lower OOP medical expenses, there is no association between the degree of full implementation of the policy and its effect on health care use.

We investigated this issue formally in a regression framework (Appendix S3) and found no significant relationship between the degree of implementation of the policy and its effect on health care utilisation once accounting for other elements affecting policy implementation.

### 5.5 Heterogeneous effects

We now explore the policy impact among the 50% poorest and the 50% richest 13It was not possible to split the sample by income groups more finely given the limited number of household in some districts (e.g., some districts do not include individuals of all quartiles, because the latter are defined at the national level). households (Table 5). 14Note that yearly deflated total household expenditure per adult equivalent was 4.1 times greater among the 50% richest (US$ 377) than for the 50% poorest (US$ 92) in 2004 and that health care use was 54% for the 50% poorest and 59% among the 50% richest in 2004 (Appendix S4). We find that there was no increase in health care use for either group (Panel A). However, we find that removing fees in government and mission facilities led the 50% richest away from private providers, with an increase in the probability of using a government or mission facility by about 18 percentage points (Panel B). Finally, the policy resulted in a similar relative decrease in OOP expenses for the rich and the poor (Panel C). Because the rich use and spend more on health, for any episode of illness, the policy resulted in a higher reduction of OOP medical expenses for the 50% richest in absolute terms (US$3.21) compared to the 50% poorest (US$1.07). 15Calculations based on applying the % reduction in OOP estimated in Table 5 to the deflated OOP medical expenses incurred in 2004. Once adjusting for the fact that the fraction of the population 16When accounting for the likelihood of being sick (equal to about 10% in the two groups), we find that about 6% and 5% of individuals in the richest and poorest group, respectively, sought care in 2004 in the past 2 weeks. that has an episode of illness and that seeks care is different between the two income groups, we find that, on average, the policy change represented a yearly government transfer worth about US$4.47 and US$1.13 to each individual of the richest and poorest group, respectively.

**Table 5:** Effect of the policy by income groups

	50% poorest			50% richest		
	(1)	(2)	(3)	(4)	(5)	(6)
Panel A: % seeking care						
Estimated effect	**−0.011**	**0.031**	**0.035**	**−0.022**	**0.013**	**0.009**
CI	[−0.31, 0.32]	[−0.31, 0.32]	[−0.31, 0.32]	[−0.182, 0.149]	[−0.182, 0.149]	[−0.182, 0.149]
*N* treated	53	54	51	51	48	51
*N* placebo	18	16	15	18	16	15
Panel B: % choosing government or mission provider						
Estimated effect	**0.007**	**0.059**	**0.064**	**0.034**	**0.181**	**0.153**
CI	[−0.11, 0.08]	[−0.11, 0.05]	[−0.10, 0.05]	[−0.22, 0.16]	[−0.22, 0.15]	[−0.16, 0.15]
*N* treated	47	47	47	50	50	49
*N* placebo	17	15	14	18	16	15
Panel C: Ln(oop)						
Estimated effect	**−1.573**	**−1.694**	**−1.720**	**−2.298**	**−2.413**	**−2.624**
*% change*	*−79%*	*−82%*	*−82%*	*−90%*	*−91%*	*−93%*
CI	[−2.59, 3.08]	[−2.05, 3.08]	[−2.05, 3.08]	[−2.01, 1.79]	[−2.01, 1.79]	[−2.01, 1.79]
*N* treated	49	50	50	50	50	51
*N* placebo	18	16	15	17	15	14
Panel D: % buying drugs in the private sector						
Estimated effect	**0.007**	**0.009**	**0.010**	**−0.261**	**−0.271**	**−0.120**
CI	[−0.08, 0.12]	[−0.08, 0.12]	[−0.08, 0.12]	[−0.32, 0.16]	[−0.31, 0.16]	[−0.31, 0.11]
*N* treated	52	49	47	45	49	45
*N* placebo	18	16	15	18	16	15

*Note*. Effects of the policy are reported in bold and percentage change are reported in italics. The 90% CI in brackets reports the 5th and 95th percentile of the placebo test distribution. Specifications (1) and (4) are estimated using all 18 urban districts as control districts. Specifications (2) and (5) exclude two districts where more than 50% of the population was reported to have benefited from free care in 2006. Specifications (3) and (6) exclude the previous two districts and a third one where more than 10% of the population sought care in a rural district in 1998.

## 6 ROBUSTNESS CHECKS

### 6.1 Match quality

The validity of the synthetic control method partly relies on the quality of the pretreatment match and the extent to which the synthetic districts are able to reproduce the pre‐intervention outcomes. Appendix S5, which shows the 54 plots presenting the pre‐intervention outcomes for each district and their twin, suggests that some synthetic controls were not able to reproduce the pretrend outcomes perfectly. In turn, this could lead to poor estimates of the policy effect. To account for quality match in the estimation of the national policy effect, we weighted each district effect by the inverse of the logarithm of the RMSPE, effectively giving a higher weight to the closely matching synthetic districts (see Panel A of Table 6). We find the same results as before.

**Table 6:** Effects accounting for pretreatment match quality

	Seek care	Public facility	OOP medical expenses	Private drug seller
Panel A: Weighted average by the logarithm of the inverse of the RMSPE				
Estimated effect	0.007	0.016	−2.177	−0.062
Panel B: Restricting the sample to high‐quality matches				
Number of perfect matches	9	19	13	28
National effect weighted by district size	0.042	0.024	−2.085	−0.080

*Note*. Perfect‐quality matches means that RMSPE < 0.01 for binary outcomes and <0.001 for ln(OOP). RMSE = root mean squared prediction error.

Next, we estimate the national effect by including only districts with high‐quality matches. We find very similar results except for health care use, where, based on only nine high‐quality district twins, the estimated policy effect seems slightly higher.

### 6.2 Micro data‐level analysis

As an alternative estimation approach, we use micro‐level data and combine DiD with propensity score matching (Heckman, Ichimura, & Todd, 1998; Imbens, 2004) to account for the nonparallel pre‐intervention trends and perform a kernel matching over three groups: the treated and control at baseline t_0_ and the nontreated at follow up t_1_. Following Blundell and Dias (2009), the matching estimator combined with DiD (MDiD), noted *α*^*MDiD*^, is given by

$$\alpha^{\text{MDiD}}=\sum_{\mathrm{i\epsilon T}1}\left\{\left[y_{\mathrm{it}1}-\sum_{\mathrm{j\epsilon T}0}{\tilde{w}}_{\mathrm{ijt}0}^{T}\;y_{\mathrm{jt}0}\right]-\left[\sum_{\mathrm{j\epsilon C}1}{\tilde{w}}_{\mathrm{ijt}1}^{C}\;y_{\mathrm{jt}1}-\sum_{\mathrm{j\epsilon C}0}{\tilde{w}}_{\mathrm{ijt}0}^{C}\;y_{\mathrm{jt}0}\right]\;\right\}\;\omega_{i},$$

where *T*_0_, *T*_1_, *C*_0_, and *C*_1_ stand for the treatment and comparison group before and after user fee removal, and ${\tilde{w}}_{\mathrm{ijt}}^{G}$ represents the weight attributed to individual *j* in group *G* (treatment or control) and time *t* (*t*_0_, *t*_1_) when comparing with treated individual *i*.

The results obtained using DiD with matching, reported in Table 7, are similar to the ones obtained using the synthetic control method. Specifically, we find that there was no impact of user fee removal on the use of modern care. However, here, the small substitution effect (4.5 percentage points) away from the private sector is found significant at the 10% level (see Panel B, Column 6). The results also confirm the large and significant decrease in OOP expenditures because the policy led to a decrease in OOP health expenditures by 86%. Finally, the results suggest that there was no impact on the likelihood of buying drug from the private sector.

**Table 7:** Effects of user fee removal using individual‐level data

	(1) Simple DiD model			(2) Matching and DiD (MDiD)		
	Diff (T‐C) baseline	Diff (T‐C) follow‐up	DiD	Diff (T‐C) baseline	Diff (T‐C) follow‐up	DiD
Panel A: % seeking care						
Estimated effect	−0.020	0.026	**0.046**	0.023	0.059***	**0.036**
*SE*	(0.025)	(0.034)	(0.034)	(0.023)	(0.04)	(0.045)
*N*	10,295	7,841	18,136	9,711	6,859	16,570
Panel B: % choosing government or mission provider						
Estimated effect	0.036	0.110***	**0.075** ***	0.026	0.072***	**0.045** *
*SE*	(0.024)	(0.026)	(0.024)	(0.023)	(0.016)	(0.026)
*N*	5,975	4,817	10,792	5,685	4,259	9,944
Panel C: Ln(oop)						
Estimated effect	−0.771**	−2.755***	**−1.985** ***	−0.195	−2.141***	**−1.946** ***
*% change*	*−53.8%*	*−93.7%*	*−86.3%*	*−17.7%*	*−88.2%*	*−85.7%*
*SE*	(0.370)	(0.467)	(0.367)	(0.161)	(0.165)	(0.374)
*N*	8,620	6,806	15,426	8,144	6,016	14,170
Panel D: % buying drugs in the private sector						
Estimated effect	−0.139***	−0.168***	**−0.029**	−0.052	−0.077**	**−0.025**
*SE*	(0.052)	(0.050)	(0.038)	(0.046)	(0.036)	(0.043)
*N*	8,589	6,791	15,380	8,127	6,003	14,130

*Note*. Effects of the policy are reported in bold and percentage change are reported in italics. The propensity score was estimated using the same covariates at the individual level than the ones used in the synthetic control. Survey sampling weights are used. SE clustered at the district level in bracket. Estimated presented in (2) are based on propensity score matching using Epanechnikov kernel weights. DiD = difference‐in‐differences

* Statistically significant at the 1% statistical significance level.

** Statistically significant at the 5% significance level.

*** Statistically significant at the 10% significance level.

### 6.3 Alternative approach to apply the synthetic control method with multiple treated units

**Figure 4:**
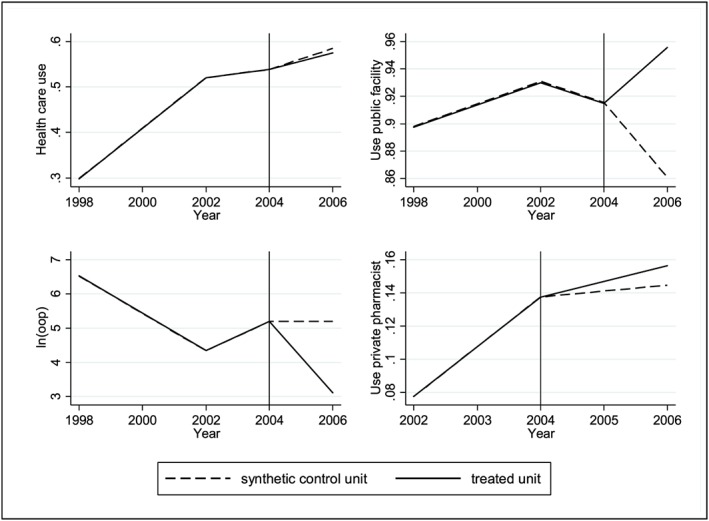
Synthetic control and treated unit trends by outcome and donor pools. The graphs are based on Specification (2) but graphs for Specifications (1) and (3) lead to similar pre‐intervention trends for the synthetic control and treated units [Colour figure can be viewed at http://wileyonlinelibrary.com]

Here, we use an alternative method to estimate a unique national effect from multiple treated units. More specifically, we aggregated outcomes and covariates from the 54 treated units to create a single treated unit and then create a synthetic rural unit with the 18 urban districts. We can see from Figure 4 that the pre‐intervention trend of the synthetic rural unit perfectly overlaps the one from the single treated unit. The results we find (Table 8) are very similar to the ones based on the 54 treated units. Specifically, we find that the policy had no effect on health care use or on the health provider chosen. However, the result confirms that the policy reduced OOP medical spending by at least 85%. Note that although collapsing all 54 treated units into one average treated unit reduces sampling error and hence leads to a lower RMSPE (see Figure 4), it does not account for the fact that each rural district has its own specificity and that taking the average outcomes in those districts could prevent from creating a counterfactual that closely accounts for time variant and invariant unobserved characteristics.

**Table 8:** Results from the synthetic control method with a single treated unit

	(1)	(2)	(3)
Seek care	−0.018	−0.010	−0.013
	[−0.16, 0.13]	[−0.16, 0.13]	[−0.16, 0.13]
Chose government or mission provider	0.029	0.094	0.088
	[−0.19, 0.12]	[−0.19, 0.12]	[−0.17, 0.12]
OOP medical expenses	−1.895	−2.078	−2.086
	[−1.99, 1.56]	[−1.00, 1.56]	[−1.00, 1.56]
*% change*	*−85.0%*	*−87.5%*	*−87.6%*
Purchase of private drugs	−0.036	0.012	0.014
	[−0.24, 0.19]	[−0.24, 0.19]	[−0.24, 0.19]

*Note*. 90% CI in brackets. Specification (1) is estimated using all 18 urban districts as control districts. Specification (2) excludes two districts where more than 50% of the population was reported to have benefited from free care in 2006. Specification (3) excludes the previous two districts and a third one where more than 10% of the population sought care in a rural district in 1998. OOP = out‐of‐pocket.

## 7 DISCUSSION AND CONCLUSIONS

Our findings indicate that the abolition of user fees in rural Zambia in 2006 did not change the probability of seeking modern care in the population. This seems to contradict the conclusions of previous studies that suggested substantial increases in the volume of outpatient visits recorded in routine data (Lagarde et al., 2012; Masiye et al., 2010). Setting aside the methodological problems associated with these past studies, 17These earlier studies did not address the biases associated with reporting inaccuracies by facilities or any changes in health‐seeking behaviours over time that were unrelated to the policy change. our results suggest that part of this increase may have come from richer patients previously seeking care in the private sector. Moreover we find that there was a large and positive effect of the policy on OOP expenditures, which decreased by nearly 90% in the population, indicating that there was a positive effect of the policy on financial protection. Due to unequal medical spending between richer and poorer groups, we find that although the reduction was similar in relative terms for both groups, in absolute terms, the policy change benefited the richest, through an income transfer per medical visit of US$3.2 for them versus US$1.1 for the poorest. Finally, we find that despite its relative lack of preparation, the policy change did not lead to drug stock‐outs in intervention areas. This result may be linked to reports of widespread shortage of drugs across the country in 2006, before the policy took place (Carasso et al., 2010).

Our results echo other recent robust empirical studies of the effects of abolishing user fees on the demand for curative care in other low‐income settings (King et al., 2009; Mohanan et al., 2014), although one study from Ghana found a slightly more encouraging increase of the demand by 3.7 percentage points (Powell‐Jackson et al., 2014). These results are at odds with the recent experimental literature on the price effects of the demand for preventive health products and services (Dupas & Miguel, 2016). There are three main explanations for the lack of effectiveness of the policy change on the demand for curative health care services.

A first explanation could be that the scrapping of official charges was replaced by the introduction (or increase) of informal payments, as suggested to have been the case in Uganda (Xu et al., 2006). However, to the extent that individuals report informal charges, our results reject this explanation, because we see an important reduction in OOP medical expenses for those visiting publicly funded health facilities.

Another potential explanation is that the demand for curative health care is price inelastic. This might have been possible because the level of fees was particularly low, and because the demand was primarily determined by other factors, such as indirect financial costs. This is supported by an analysis of the determinants of health‐seeking behaviours before 2006 (see Appendix S6). Although the results show a positive association between income and the probability to seek care, the magnitude of this effect is quite small, 18An increase in one point in the logarithm of deflated total household expenditures in adult equivalent (similar to an increase in one tercile in the income distribution) is associated with an increase in health care use by only 4.4 percentage points and a decrease in the likelihood of going to a public health facility by 3.3 percentage points. even in rural districts and among the 50% poorest households. In addition, data from LCMS 1998 suggest that indirect costs to accessing care were as important as OOP expenses. Even 7 years after fees were abolished nationally, 11% of patients in rural areas reported catastrophic health expenditures 19Defined as 40% of household nonfood expenditures. mostly because of transportation costs, which represented 73% of these costs (Masiye, Kaonga, & Kirigia, 2016).

A final explanation is that changes occurred over the period of study, which changed individuals' valuation of health care services. The main suspicion is that quality of care in rural areas deteriorated over the period, possibly partly as a result of the policy change. First, lack of planning and delays in providing adequate funding may have led to deterioration of the quality of services, as suggested before. The uncompensated loss of revenue from the removal of user fees can have important consequences at facility level where a significant share of fee revenue is retained to finance a proportion of staff income (Carasso et al., 2010). The policy change may also have exacerbated problems of motivation and shortages of staff in rural areas (Picazo & Zhao, 2009). Because of the substitution away from private‐for‐profit providers and a possible increase in the intensity of health care use, waiting times in health facilities may have increased. Unfortunately, in the absence of data on the quality of care, we cannot disentangle whether the absence of effect on utilisation is mostly explained by the price inelasticity of the demand or a combination of an increase in demand due to the price effect and a downward shift in demand resulting from a poorer quality of care.

In addition to the challenge to identify clearly the reasons behind the lack of effect found on the demand for health care, this study suffers from several limitations.

A first limitation relates to the small number of control units. As a result, the confidence intervals estimated from the placebo effects distribution are relatively large and might preclude a more precise estimation of effects. Still, the upper bound confidence interval from the placebo tests is close to the one obtained with MDiD presented as a robustness check. 20MDiD 90% upper bound CI = 0.11. Besides, the synthetic control method remains superior to MDiD estimator because, unlike the propensity score matching conducted on 2004 data only, the synthetic districts are created using multiple pre‐intervention periods, allowing us to account for unobserved heterogeneity that varies over time.

Another limitation relates to the messy definition and implementation of the policy, resulting in the potential contamination of the control units in our dataset, which may have led to problems in estimating the effects of the policy (see Section 4.2). We investigated this issue further in Appendix S7. We found additional support for the idea that the effect on health provider choice could be underestimated given that, for this outcome, contaminated placebo districts were given a higher weight in the synthetic control. However, this was not the case for the other outcomes. Besides, Appendix S7 also shows that the contamination issue was not the reason for our larger confidence intervals because the effect of the policy was not stronger in contaminated urban districts.

Finally, the challenges to implement the policy could provide an explanation of the absence of short‐term effect on utilisation. However, as highlighted before, a study using longitudinal health facility routine data (Lagarde et al., 2012) showed that utilisation peaked 6 months after the implementation. This suggests that the timeline we use is probably ideal to capture an effect on utilisation. 21Unfortunately, the later scale‐up of the policy does not allow the identification of medium‐ or long‐term effects.


In January 2007, user fees were removed in all the facilities located in the peri‐urban areas of the 18 districts where fees had not yet been abolished. 22The remit of this scale‐up, which could not be well identified in the household survey data due to the absence of geographic coordinates, prevented the use of the 2010 LCMS wave to assess the longer term consequences of the policy. Later, in January 2012, the policy was extended to all remaining areas. A study looking at the financial protection conferred by the free care policy found that 29.9% and 45% of patients in public rural and urban health centres respectively incurred some expenditures (Masiye et al., 2016). In a follow‐up study using the same data, Masiye and Kaonga (2016) conclude that in 2014, “despite the removal of user fees in public primary healthcare in Zambia, access to healthcare is highly dependent on an individual's socio‐economic status, illness type and region of residence.” Together with our findings, this suggests that user fee removal may not necessarily be the silver bullet to move towards greater access for the poorer population and quicker move towards universal coverage. Evidence from several settings also point to the potential disruptive effects of free care policies on health systems (e.g., drug shortages, staff dissatisfaction, and insufficient funding) and therefore the need to prepare, plan, and introduce complementary measures to ensure a more positive outcome (Ridde, Robert, & Meessen, 2012)

The debate over whether low‐ and middle‐income countries should charge their populations for using health care services has been highly contentious for several decades. This study suggests important and maybe counter‐intuitive lessons for policy‐makers, with regards to the immediate equity effects of removing user charges. If removing fees does not increase utilisation of services, in particular of the poorest, but effectively reduces OOP medical expenses of those using the services, then the beneficiaries of the policy change are those individuals already using services. The conclusion that user fee removal in Zambia was primarily benefiting the richer groups echoes a typical problem of policies promoting universal access to services in settings where initial inequalities are large and there are multiple barriers to accessing services for the poor (Gwatkin & Ergo, 2011). It would be interesting to know whether removing these other barriers (e.g., through financial or nonfinancial incentives) while maintaining user fees would be more effective than removing fees.

## Supporting information

Appendix S1: Proportion living in rural areas in urban and rural districts
Appendix S2: Placebo effects
Appendix S3: Determinants of effects
Appendix S4: Descriptive statistics for 50% poorest and 50% richest in 2004 (*n* = 72)
Appendix S5: Pre‐intervention match quality
Appendix S6: Determinants of health seeking behaviours prior user fee removal
Appendix S7: Potential effects of contamination on estimated impact and confidence intervals
