# Supplementary material for: How effective and fair is user fee removal? Evidence from Zambia using a pooled synthetic control
Source: Health Econ. 2017 Oct 16;27(3):493–508. doi: 10.1002/hec.3589 (PMC5900920; doi:10.1002/hec.3589)
Supplement: Supplementary file 1 — Appendix S1: Proportion living in rural areas in urban and rural districts Appendix S2: Placebo effects Appendix S3: Determinants of effects Appendix S4: Descriptive statistics for 50% poorest and 50% richest in 2004 (n = 72) Appendix S5: Pre‐intervention match quality Appendix S6: Determinants of health seeking behaviours prior user fee removal Appendix S7: Potential effects of contamination on estimated impact and confidence intervals [file HEC-27-493-s001.docx]

**Appendix 1:** **Proportion living in rural areas in urban and rural districts**

**
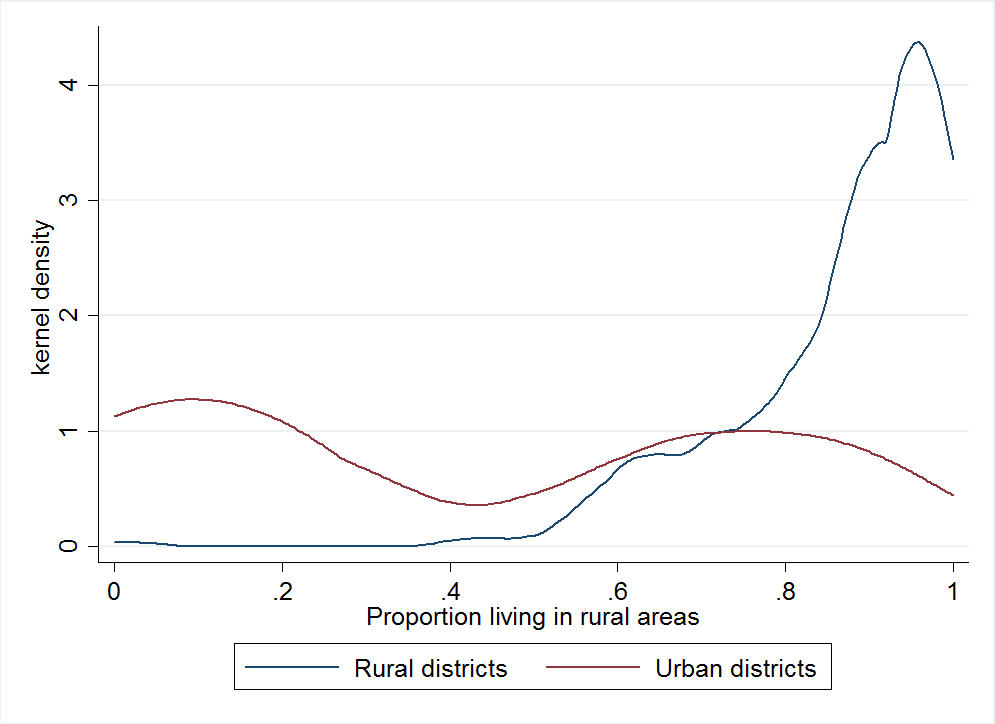
**

**Appendix 2: Placebo effects**

| 1. 18 controls | 1. 16 controls | 1. 15 controls |
| --- | --- | --- |
|  |  |  |
| Notes: The graph displays in black the national effect averaged across treated (rural) districts and in grey the effect of the policy in each control (urban) district. | | |

**Appendix 3: Determinants of effects**

|  | (1) | | (2) | | (3) | | (4) | |
| --- | --- | --- | --- | --- | --- | --- | --- | --- |
|  | Effect on seek care | | Effect on public facility use | | Effect on ln(oop) | | Effect on drugs bought in private | |
|  | coef | se | coef | se | coef | se | coef | se |
| Degree of implementation | 0.028 | (0.164) | 0.216*** | (0.067) | -4.399*** | (0.926) | -0.300 | (0.216) |
| Median distance to facility | -0.000 | (0.004) | 0.002 | (0.003) | -0.030 | (0.031) | 0.005 | (0.004) |
| Median income | 0.016 | (0.047) | 0.011 | (0.022) | 0.303 | (0.513) | 0.035 | (0.051) |
| Median age | -0.028 | (0.019) | 0.004 | (0.007) | 0.170 | (0.160) | 0.002 | (0.021) |
| Population density | -0.000 | (0.001) | -0.000 | (0.001) | 0.019 | (0.014) | 0.001 | (0.001) |
| Rural location | -0.494** | (0.198) | -0.000 | (0.137) | 2.110 | (2.204) | 0.100 | (0.264) |
| Copperbelt (ref : Central) | 0.059 | (0.077) | 0.020 | (0.053) | 0.543 | (0.454) | 0.017 | (0.075) |
| Eastern | 0.150* | (0.085) | -0.069 | (0.062) | -0.455 | (1.099) | 0.016 | (0.091) |
| Luapula | 0.099 | (0.084) | -0.053 | (0.064) | 0.429 | (0.722) | 0.118* | (0.070) |
| Lusaka | -0.026 | (0.066) | -0.053 | (0.054) | 0.753 | (0.520) | 0.235*** | (0.063) |
| Northern | 0.039 | (0.065) | -0.045 | (0.056) | 0.901 | (0.680) | 0.162*** | (0.051) |
| Northern Western | 0.214*** | (0.071) | -0.002 | (0.058) | -0.553 | (0.635) | 0.100 | (0.076) |
| Southern | 0.081 | (0.074) | -0.003 | (0.057) | 0.540 | (0.705) | 0.120 | (0.074) |
| Western | 0.072 | (0.089) | -0.032 | (0.076) | 0.398 | (0.865) | 0.084 | (0.100) |
| Constant | 0.707 | (0.785) | -0.245 | (0.380) | -8.451 | (8.317) | -0.529 | (0.814) |
| Observations | 51 |  | 53 |  | 49 |  | 49 |  |
| R-squared | 0.309 |  | 0.306 |  | 0.604 |  | 0.405 |  |

Notes: Estimates are adjusted by district size. * Statistically significant at the 1% statistical significance level ** at the 5% significance level and *** at the 10% significance level.

**Appendix 4: Descriptive statistics for 50% poorest and 50% richest in 2004 (n=72)**

|  | 50% poorest | | 50% richest | |
| --- | --- | --- | --- | --- |
|  | Mean | SE | Mean | SE |
| Seeking modern care (%) | 0.542 | 0.018 | 0.594 | 0.018 |
| Went to a public health facility (%) * | 0.917 | 0.014 | 0.896 | 0.016 |
| Deflated out-of-pocket medical expenditures (USD) * | 1.349 | 0.208 | 3.569 | 0.742 |
| Bought drugs from the private sector (%) * | 0.027 | 0.009 | 0.044 | 0.009 |
| Yearly deflated total household expenditure per adult equivalent (USD) | 92.424 | 1.709 | 376.782 | 8.987 |
| Distance to facility (km) | 6.145 | 0.450 | 4.543 | 0.355 |
| Household size | 6.696 | 0.123 | 7.909 | 0.137 |
| Living in rural location (%) | 0.779 | 0.033 | 0.595 | 0.034 |

Note: All values are representative at the national level as the district panel data was constructed based on sampling weights. *of the proportion of individuals seeking modern care

**Appendix 5: Pre-intervention match quality**

| Seek care   | |  |  |
| --- | --- | --- | --- |
| Choice of government or mission facilities   | | | |
| ln(OOP)   | | | |
| Purchase of drugs in a private pharmacy | |  |  |

**Appendix 6: Determinants of health seeking behaviours prior user fee removal**

|  | **Sought care** | | | | | | | |  | | | | **Chose a government or mission facility** | | | | | | | | | | | | | | | | |  |
| --- | --- | --- | --- | --- | --- | --- | --- | --- | --- | --- | --- | --- | --- | --- | --- | --- | --- | --- | --- | --- | --- | --- | --- | --- | --- | --- | --- | --- | --- | --- |
|  | (1)  Whole sample | | (2)  Rural districts | | (3)  50% poorest | | | |  | | | | (4)  Whole sample | | | | | | (5)  Rural districts | | | | | (6)  50% poorest | | | | | |  |
|  |  |  |  |  |  | |  | |  | | | |  | | |  | | |  | | |  | |  | | |  | | |  |
|  | coef | se | coef | se | | coef | se | | |  | | coef | | | se | | | coef | | | se | | | coef | | | se | | |  |
| Age | -0.001** | (0.000) | -0.0004 | (0.000) | | -0.001*** | | (0.000) | | |  | | | -0.0005** | | | (0.000) | | | -0.0003 | | | (0.000) | | | -0.001** | | (0.000) | | |
| Male | 0.005 | (0.009) | 0.006 | (0.011) | | 0.012 | | (0.012) | | |  | | | -0.023*** | | | (0.007) | | | -0.021** | | | (0.009) | | | -0.025** | | (0.010) | | |
| Household size | 0.002 | (0.001) | 0.001 | (0.002) | | 0.000 | | (0.002) | | |  | | | 0.001 | | | (0.001) | | | 0.002* | | | (0.001) | | | 0.002 | | (0.002) | | |
| **Log deflated expenditures in adult equivalent** | **0.044***** | **(0.005)** | **0.041***** | **(0.006)** | | **0.030***** | | **(0.009)** | | |  | | | **-0.033***** | | | **(0.005)** | | | **-0.011**** | | | **(0.006)** | | | **-0.022***** | | **(0.008)** | | |
| Distance to facility (std) | -0.049*** | (0.005) | -0.054*** | (0.005) | | -0.046*** | | (0.005) | | |  | | | -0.028*** | | | (0.006) | | | -0.030*** | | | (0.007) | | | -0.034*** | | (0.008) | | |
| Rural location | -0.004 | (0.011) | -0.022 | (0.014) | | 0.012 | | (0.014) | | |  | | | 0.060*** | | | (0.008) | | | 0.043*** | | | (0.011) | | | 0.015 | | (0.011) | | |
| Copperbelt (ref : Central) | 0.022 | (0.017) | 0.039 | (0.030) | | 0.037 | | (0.023) | | |  | | | -0.022 | | | (0.016) | | | 0.011 | | | (0.022) | | | 0.044** | | (0.022) | | |
| Eastern | 0.036* | (0.019) | 0.053** | (0.022) | | 0.045* | | (0.024) | | |  | | | 0.050*** | | | (0.016) | | | 0.048*** | | | (0.017) | | | 0.057** | | (0.022) | | |
| Luapula | 0.007 | (0.018) | 0.016 | (0.021) | | -0.011 | | (0.023) | | |  | | | 0.046*** | | | (0.015) | | | 0.031* | | | (0.017) | | | 0.055*** | | (0.021) | | |
| Lusaka | 0.063*** | (0.018) | 0.151*** | (0.025) | | 0.059** | | (0.027) | | |  | | | -0.029 | | | (0.019) | | | -0.050** | | | (0.025) | | | -0.001 | | (0.029) | | |
| Northern | -0.042** | (0.018) | -0.042** | (0.021) | | -0.032 | | (0.022) | | |  | | | 0.024 | | | (0.018) | | | -0.008 | | | (0.021) | | | 0.052** | | (0.024) | | |
| Northern Western | 0.133*** | (0.020) | 0.171*** | (0.023) | | 0.134*** | | (0.025) | | |  | | | 0.038** | | | (0.017) | | | 0.068*** | | | (0.017) | | | 0.031 | | (0.024) | | |
| Southern | 0.045** | (0.021) | 0.078*** | (0.023) | | 0.058** | | (0.027) | | |  | | | 0.029* | | | (0.017) | | | 0.008 | | | (0.021) | | | 0.016 | | (0.025) | | |
| Western | 0.100*** | (0.020) | 0.100*** | (0.023) | | 0.117*** | | (0.024) | | |  | | | 0.066*** | | | (0.016) | | | 0.053*** | | | (0.017) | | | 0.076*** | | (0.021) | | |
| 2002 (ref :1998) | 0.205*** | (0.011) | 0.228*** | (0.015) | | 0.210*** | | (0.014) | | |  | | | 0.031*** | | | (0.011) | | | 0.027* | | | (0.014) | | | 0.011 | | (0.015) | | |
| 2004 | 0.242*** | (0.011) | 0.243*** | (0.014) | | 0.261*** | | (0.014) | | |  | | | 0.043*** | | | (0.011) | | | 0.016 | | | (0.013) | | | 0.023 | | (0.014) | | |
| Constant | -0.288*** | (0.073) | -0.245*** | (0.088) | | -0.125 | | (0.121) | | |  | | | 1.261*** | (0.064) | | | | | 0.999*** | | | (0.079) | 1.162*** | | | | | (0.108) | |
|  |  |  |  |  | |  | |  | | |  | | |  |  | | | | |  | | |  | |  | | | |  | |
| Observations | 22,624 |  | 14,058 |  | | 12,239 | |  | | |  | | | 11,641 |  | | | | | 7,213 | | |  | | 5,755 | | | |  | |
| R-squared | 0.067 |  | 0.083 |  | | 0.068 | |  | | |  | | | 0.047 |  | | | | | 0.033 | | |  | | 0.030 | | | |  | |

Note: Standard errors in parentheses ; *** p<0.01, ** p<0.05, * p<0.1

**Appendix 7: Potential effects of contamination on estimated impact and confidence intervals**

A: Relationship between the degree of contamination and the average weight of the placebo districts

B: Relationship between the degree of contamination and the effect of the policy in the placebo districts
